# Long-term fertilization coupled with rhizobium inoculation promotes soybean yield and alters soil bacterial community composition

**DOI:** 10.3389/fmicb.2023.1161983

**Published:** 2023-05-18

**Authors:** Wanling Wei, Dawei Guan, Mingchao Ma, Xin Jiang, Fenliang Fan, Fangang Meng, Li Li, Baisuo Zhao, Yubin Zhao, Fengming Cao, Huijun Chen, Jun Li

**Affiliations:** ^1^Institute of Agricultural Resources and Regional Planning, Chinese Academy of Agricultural Sciences, Beijing, China; ^2^Laboratory of Quality & Safety Risk Assessment for Microbial Products (Beijing), Ministry of Agriculture, Beijing, China; ^3^Soybean Research Institute, Jilin Academy of Agricultural Sciences, Jilin, China

**Keywords:** fertilization, rhizobium inoculation, bacterial community, rhizosphere soil, soybean

## Abstract

Microbial diversity is an important indicator of soil fertility and plays an indispensable role in farmland ecosystem sustainability. The short-term effects of fertilization and rhizobium inoculation on soil microbial diversity and community structure have been explored extensively; however, few studies have evaluated their long-term effects. Here, we applied quantitative polymerase chain reaction (qPCR) and amplicon sequencing to characterize the effect of 10-year fertilizer and rhizobium inoculation on bacterial communities in soybean bulk and rhizosphere soils at the flowering–podding and maturity stages. Four treatments were examined: non-fertilization control (CK), phosphorus and potassium fertilization (PK), nitrogen and PK fertilization (PK + N), and PK fertilization and *Bradyrhizobium japonicum* 5821 (PK + R). Long-term co-application of rhizobium and PK promoted soybean nodule dry weight by 33.94% compared with PK + N, and increased soybean yield by average of 32.25%, 5.90%, and 5.00% compared with CK, PK, and PK + N, respectively. The pH of PK + R was significantly higher than that of PK and PK + N at the flowering–podding stage. The bacterial abundance at the flowering–podding stage was positively correlated with soybean yield, but not at the maturity stage. The significant different class Gemmatimonadetes, and the genera *Gemmatimonas*, and *Ellin6067* in soil at the flowering–podding stage were negatively correlated with soybean yield. However, the bacterial community at class and genus levels at maturity had no significant effect on soybean yield. The key bacterial communities that determine soybean yield were concentrated in the flowering–podding stage, not at maturity stage. Rhizosphere effect, growth period, and treatment synergies resulted in significant differences in soil bacterial community composition. Soil organic matter (OM), total nitrogen (TN), pH, and available phosphorus (AP) were the main variables affecting bacterial community structure. Overall, long-term co-application of rhizobium and fertilizer not only increased soybean yield, but also altered soil bacterial community structure through niche reconstruction and microbial interaction. Rhizobium inoculation plays key role in reducing nitrogen fertilizer application and promoting sustainable agriculture practices.

## 1. Introduction

Chemical fertilizer application is a common strategy for enhancing soil fertility and crop yield in agricultural production. Effective fertilization can promote soil ecosystem health. However, irrational fertilization can lead to a series of environmental problems, including soil biodiversity and productivity reduction (Guo et al., 2010; Yang et al., 2018), environmental pollution (Probert et al., 1998; Drijber et al., 2000), and soil acidification (Ahmad et al., 2013; Xiao et al., 2021; Xie et al., 2021). At present, measures such as fallowing, organic substitution (Dai et al., 2019; Ji et al., 2020; Zhang et al., 2021), green manure planting (Zhang et al., 2017; Ma et al., 2021), and biofertilizer promotion (Wu et al., 2005; Castro et al., 2020; Zhou et al., 2021) have been implemented in agriculture to reduce the detrimental impacts of fertilization. Biofertilizers have become a research hotspot because of their environmental friendliness and effectiveness. They can activate fixed nutrients in the soil, stimulate growth and absorption by the root system, and reduce fertilization rates with the corresponding nutrient elements (Abd-Alla et al., 2014; Koskey et al., 2017). With such soil adjustments, beneficial bacteria can be increased, harmful bacteria eliminated, and crop diseases reduced (Igiehon and Babalola, 2017; Schütz et al., 2018). Soybean rhizobia are biofertilizers and facilitate nitrogen fixation and emission reduction (Soumare et al., 2020).

The Gram-negative soil bacterium rhizobium is widely distributed and can stimulate legumes to produce root nodules and symbiotic nitrogen fixation (Ditta et al., 1980; Quandt and Hynes, 1993; Han et al., 2020). Rhizobium has positive effects on crop growth and nutrient absorption, including promoting the absorption of phosphorus and iron, and enhancing the production of plant hormones that promote crop growth. They can additionally enlarge the abundance of beneficial microorganisms, minimize the prevalence of pests and diseases, and expand crop yield (Zhuang et al., 2007; Ahemad and Kibret, 2014). Trabelsi and Mhamdi (2013) reported that inoculation with two local rhizobia strains increased potato yield by 32% and decreased potato wireworm infestation by 56%. The plant hormone gibberellin produced by rhizobia enlarges the size of nodules in host legumes (Nett et al., 2022). Furthermore, inoculation with rhizobia can promote yields in crops, such as soybean (Ronner et al., 2016), milk vetch (Liu et al., 2022), faba bean (Pereira et al., 2019), maize (Marks et al., 2015), wheat (Yanni et al., 2016), and rice (Mehboob et al., 2009). In addition, the effect of rhizobia inoculum alone is reportedly weaker than that of rhizobia combined with other strains or chemical fertilizer. The nodulation rate and yield of soybean could be improved by co-inoculation of *Bradyrhizobium japonicum* and *Azospirillum brasilense* (Hungria et al., 2013; Barbosa et al., 2021; Moretti et al., 2021). Combination of *Rhizobium* sp. and chemical fertilizer (N_15_P_15_K_15_) achieved the best peanut nutrition and production parameters (Adjile et al., 2020). Rhizobium has been demonstrated to be an environmentally friendly substitute to nitrogen fertilizer based on the positive effects above (Gitonga et al., 2021; Halwani et al., 2021; Pires et al., 2021; Kumawat et al., 2022). Although rhizobium can form symbiotic relationships with legumes, and provide a considerable number of nitrogen to plant, continuous inoculation ensures the long-term availability of nitrogen in the soil. Therefore, it is an ecologically manageable choice for enhancing agricultural soil environment (Korir et al., 2017; Thilakarathna and Raizada, 2017; Zilli et al., 2021).

Several long-term studies have reported that fertilization alters soil microbial community composition. Zhou et al. (2015) observed that 34 years of fertilization decreased bacterial diversity. Long-term fertilization enhanced bacterial abundance and modified bacterial composition in rhizosphere soil (Wang et al., 2018). Inoculation with rhizobia can alter soil microbial community structure; however, most of the current studies are based on short-term time scales. One-year inoculation of rhizobia and application of appropriate nitrogen fertilizer in the field increased the bacterial richness in the rhizosphere soil (Trabelsi et al., 2011). Furthermore, rhizobium inoculation may contribute to the rotational benefits of legumes in potato cropping systems not only by providing fixed nitrogen, but also by increasing microbial diversity and structure, potentially stimulating plant growth promoting rhizobacteria and enhancing disease control (Trabelsi et al., 2012). The effects of *Achillea millefolium* EO (Essential oils) and three different rhizobia on soybean were studied in a greenhouse experiment. The results showed that compared with the control, the bacterial colony forming units decreased after EO application and increased after inoculation with rhizobia (Turan et al., 2019).

Soybeans [*Glycine max* (L.) Merr.] are native to China and their seeds are rich in protein (64%) and oil (30%; Niwińska et al., 2020; Kumawat et al., 2022). Inoculating soybean crop soil with appropriate rhizobia can supplement a high number of effective rhizobia. This can further enhance the soil fertility and increase soybean yields (Hungria et al., 2013; Igiehon et al., 2021). Microbe is a vital indicator of soil fertility and performs an integral function in farmland ecosystem sustainability. To date, the short-term effects of fertilization and rhizobium inoculation on soil bacterial diversity and community composition have been explored extensively; however, few studies have evaluated their long-term effects. In the present study, a soybean field in Northeast China with a history of 10 years of chemical fertilization and inoculation with rhizobium was examined to determine the effects of the unique fertilization strategies on bacterial community abundance and composition at the flowering–podding and maturity stages, combined with amplicon sequencing and quantitative PCR (qPCR). We hypothesized that long-term fertilization and co-inoculation with rhizobium would alter the soil bacterial community structure. Inoculation with rhizobia would improve soybean yield by increasing nodule dry weight. Our results would provide novel insights into the effects of long-term fertilization and rhizobium inoculation on soybean yield and soil bacterial community structure, and provide a theoretical basis for microbial fertilizer development and utilization.

## 2. Materials and methods

### 2.1. Experimental site and soil sampling

Since 2011, a long-term fertilization experiment was carried out in the modern agricultural industrial technology demonstration base, located at the Jilin Academy of Agricultural Sciences, Gongzhuling County, Jilin Province, China (43° 52′ 88′′ N, 124° 80′ 55′′ E, 42 m elevation) with typical chernozem. The cropping system was an annual rain-fed rotation system and the main crop was soybean, which was planted continuously throughout the year. *Bradyrhizobium japonicum* 5821 was isolated from the soybean root nodule of Kenjiandou 28, Jiusan Farm, Nenjiang. 5 ml of rhizobia solution (concentration was 5 × 10^9^ CFU ml^−1^) was mixed with 1 kg soybean seeds. Sow 45 kg of soybean seeds per hectare. The soybean variety used was Jiyu 86.

Soil samples were collected at the flowering–podding (16 July), and maturity (29 September) stages in 2021. In this study, four treatments with three replicates were examined, and each replicate contained five random soybean plants. Namely (1) CK, non-fertilization control; (2) PK, phosphorus (75 kg P_2_O_5_ ha^−1^), and potassium fertilization (75 kg K_2_O ha^−1^); (3) PK + N, PK chemical fertilizers plus urea (60 kg N ha^−1^); and (4) PK + R, PK chemical fertilizers plus *B. japonicum* 5821. Topsoil (0–20 cm) and borrowed approximately 30 cm from the plants was collected as bulk soil. The rhizosphere soil of five plants was randomly collected, the excess soil was shaken off, and the soil close to the roots was gently collected with a brush and mixed into a composite sample. The soil sample was thoroughly homogenized by removing the weeds and gravel with a 2-mm sieve. Part of the soil samples were naturally air-dried and stored at 4 degrees, respectively, for physicochemical detection, and the rest were stored at −80 degrees for molecular experiments. Soybean nodules were collected from soybean roots at the flowering–podding stage, and the nodules were placed in an 80°C oven until reaching constant weight.

### 2.2. Soil physicochemical analysis

The ratio of soil to distilled water was 1:2.5 (weight/volume) was used to determine soil pH. Organic matter (OM) was measured according to loss on ignition of dried weight in a muffle furnace at 550°C for 6 h. CNS-2000 analyzer (LECO, St. Joseph, MI, USA) was used to estimate the TN content by burning of air-dried soil which was passed through a 0.15 mm sieve. Soil available N (AN) was measured by the diffusion plate alkaline hydrolysis method, and H_2_SO_4_ titration was used to determine its content (Zhou et al., 2019). Available phosphorus (AP) was extracted by 0.5 M NaHCO_3_ and the molybdenum blue colorimetric method was used for analysis (Olsen et al., 1954). Available potassium (AK) was extracted with 1 M ammonium acetate and determined by flame photometer (FP640, INASA, China).

### 2.3. Extraction of soil DNA and 16S rRNA gene quantitation

Soil DNA (1 g of fresh sample) was extracted using the DNeasy^®^ PowerSoil^®^ Kit (Qiagen, Hilden, Germany). The DNA concentration and purity were evaluated using a NanoDrop ND-1000 UV–Vis Spectrophotometer (Thermo Fisher Scientific, Rockwood, TN, USA) and 1% (w/v) agarose gel electrophoresis. The copy number of 16S rRNA gene (V4 fragment) was determined by qPCR using an ABI 7500 thermal cycler (Applied Biosystems, Waltham, MA, USA) with the primers 515FmodF (5′-GTGYCAGCMGCCGCGGTAA-3′) and 806RmodR (5′-GGACTACNVGGGTWTCTAAT-3′; Wang et al., 2018). The construction of reaction system and extraction of plasmids refer to Zhou et al. (2015). Three replicates of qPCR were performed in each group. The specificity of the amplified 16S rRNA gene was evaluated using melt curve with fluorescence measurement at temperatures ranging from 60 to 95°C. The parameter Ct (threshold period) received by ABI 7500 (version 1.0.6) was used to determine the copies of 16S rRNA gene (Zhou et al., 2019).

### 2.4. Amplicon sequencing of 16S rRNA gene

The purified DNA was amplified using primers 338F (5′-ACTCCTACGGGAGGCAGCAG-3′) and 806R (5′-GGACTACHVGGGTWTCTAAT-3′; Ding et al., 2020). They were sequenced on the MiSeq PE300 platform (Illumina, San Diego, CA, USA) at the Sanger Biotech Co., Ltd., Shanghai, China. In the reads of the original 16S rRNA gene, shorter sequences and those with ambiguous bases were discarded. QIIME (v. 1.9.1) was used to identify and remove chimeric and noisy sequences. Use UPARSE (v. 11) to cluster operational taxonomic units (OTUs) at 97% similarity cut-off points. A unique classification of OTUs was confirmed based on a comparison with the SILVA database (Release 138, http://www.arb-silva.de). Total potential OTUs and bacterial diversity were estimated using Mothur software (1.30.2). The original sequences were uploaded to the NCBI Sequence Read Archive under study PRJNA859097 (Chen et al., 2021; Zhang et al., 2022).

### 2.5. Statistical analysis

SPSS 24 (SPSS, Chicago, USA) was used to conduct one-way analysis of variance to analyze the differences in basic properties, bacterial abundance and diversity, and Duncan’s test at *p* < 0.05 was used to compare the significance between the soil treatments. The difference of physicochemical properties between bulk and rhizosphere soils was analyzed by *T*-test. The effects of fertilization, rhizosphere effect, and growth stage interaction on bacterial abundance and community diversity were analyzed by multiway analysis of variance (ANOVO) (Chen et al., 2021). Boxplot and regression analysis were carried out with the “*ggplot2*” package; principal coordinate analysis (PCoA) and Mantel test were proceeded by “*vegan*” package in R software (v 3.6.1). Redundancy analysis (RDA) was performed using CANOCO (version 5.0) to visualize the effects of soil physicochemical factors on bacterial OTU composition. The relationships among the soil properties, nodule dry weight, soybean yield, and bacterial community composition were detected by calculating Spearman correlation coefficients.

AMOS software (IBM^®^ SPSS^®^ Amos 26.0.0) was used to conduct structural equation modeling (SEM) to account for the direct and indirect relationships among soil main physicochemical factors, nodule dry weight, soybean yield, and bacterial community structure. The first principal component (PC1) of PCoA was used as the index of bacterial community composition, and the Shannon index was used as the index of bacterial diversity. The best fitting model was obtained based on the maximum likelihood of fit, namely, *p*-values, the goodness of Chi-square test (*χ*^2^) and fit index (GFI), and the approximate root mean square error (RMSEA; Lefcheck, 2015; Kwok et al., 2018).

## 3. Results

### 3.1. Variations in environmental factors among treatments

Long-term fertilization and inoculation with rhizobium changed the soil physicochemical properties, and soybean nodulation rate and yield (Table 1). Long-term nitrogen fertilization (PK + N) reduced the root nodule dry weight by 69.02% compared with PK, by 33.94% when compared with PK + R during the flowering–podding stage. The soybean yield was the highest in PK + R (3,025.33 kg ha^−1^). Inoculation with *B. japonicum* 5821 increased soybean yield by 737.73 kg ha^−1^ (32.25%) on average when compared with CK, by 168.66 kg ha^−1^ (5.90%) on average when compared with PK, and by 144.00 kg ha^−1^ (5.00%) on average when compared with PK + N. Long-term fertilization (PK, PK + N, PK + R) substantially decreased the soil pH, particularly in PK at flowering-podding stage and in PK+ N at maturity stage. The pH of the PK + R was notably greater than that of the PK and PK + N. Long-term application of PK+ N and PK + R significantly increased AP and AK content in soil at both stages. However, the effects of different treatments on TN and AN were inconsistent. Their contents fluctuated within a small range among different treatments, which might be related to the strong nitrogen fixation potential of the soybean roots. Additionally, at the flowering–podding stage, the pH of rhizosphere soil in all treatments was significantly higher than that of bulk soil. There was no significant difference between the bulk and rhizosphere contents of OM and AN in all treatments. Except PK + N, AK in other treatments was significantly greater in the rhizosphere than in the bulk soil. At the maturity stage, OM and AK in rhizosphere soil were significantly higher than that in bulk soil under fertilization conditions (*p* < 0.05). TN in bulk soil was significantly lower than that in rhizosphere soil except nitrogen fertilizer treatment.

**Table 1:** Properties of the bulk soil and rhizosphere samples from soybean cultivated at the flowering–podding stage and maturity stage under different fertilization levels.

Treatments		CK	PK	PK + N	PK + R
Flowering-podding stage					
pH	Bulk soil	5.96 ± 0.02a	5.47 ± 0.01d	5.66 ± 0.01c	5.75 ± 0.01b
	Rhizosphere	6.16 ± 0.02a^*^	5.61 ± 0.03d^*^	5.85 ± 0.01c^*^	5.99 ± 0.01b^*^
OM (g kg^−1^)	Bulk soil	28.92 ± 0.67a	29.68 ± 1.31a	30.85 ± 1.35a	30.50 ± 1.51a
	Rhizosphere	28.55 ± 1.74a	28.38 ± 0.29a	30.81 ± 1.45a	29.89 ± 0.82a
TN (g kg^−1^)	Bulk soil	1.71 ± 0.04ab	1.66 ± 0.03b	1.75 ± 0.04a	1.67 ± 0.06ab
	Rhizosphere	2.03 ± 0.08a	1.76 ± 0.13b	1.91 ± 0.12ab	1.88 ± 0.09ab^*^
AN (mg kg^−1^)	Bulk soil	122.10 ± 4.70ab	118.48 ± 3.30b	125.14 ± 1.08a	116.30 ± 3.03b
	Rhizosphere	125.37 ± 2.67a	120.84 ± 2.94a	127.90 ± 5.66a	124.99 ± 5.13a
AP (mg kg^−1^)	Bulk soil	21.43 ± 0.60c	78.27 ± 1.11a^*^	63.93 ± 1.59b^*^	64.60 ± 0.69b
	Rhizosphere	20.80 ± 1.47d	67.70 ± 0.70a	48.97 ± 0.60c	63.57 ± 1.55b
AK (mg kg^−1^)	Bulk soil	126.15 ± 1.32c	182.73 ± 4.06b	199.58 ± 6.50a	204.97 ± 3.75a
	Rhizosphere	132.59 ± 2.70c^*^	238.94 ± 7.65a^*^	197.34 ± 2.60b	242.18 ± 3.38a^*^
Nodule dry weight (g)		1.48 ± 0.06ab	1.98 ± 0.34a	1.17 ± 0.44b	1.57 ± 0.06ab
Maturity stage					
pH	Bulk soil	6.24 ± 0.03a^*^	6.02 ± 0.01b^*^	5.85 ± 0.01c	6.02 ± 0.00b
	Rhizosphere	5.93 ± 0.02b	5.95 ± 0.01ab	5.87 ± 0.02c	5.98 ± 0.03a
OM (g kg^−1^)	Bulk soil	30.09 ± 0.75ab	29.06 ± 0.17c	30.76 ± 0.08a	29.86 ± 0.46bc
	Rhizosphere	31.48 ± 0.59ab	30.78 ± 0.74b^*^	32.10 ± 0.44a^*^	32.49 ± 0.63a^*^
TN (g kg^−1^)	Bulk soil	1.56 ± 0.03a	1.49 ± 0.02a	1.45 ± 0.28a	1.56 ± 0.03a
	Rhizosphere	1.68 ± 0.01a^*^	1.72 ± 0.06a^*^	1.71 ± 0.04a	1.70 ± 0.03a^*^
AN (mg kg^−1^)	Bulk soil	138.35 ± 3.49a	140.31 ± 3.31a	140.68 ± 5.70a	144.34 ± 4.81a
	Rhizosphere	142.72 ± 2.62a	150.38 ± 3.60a^*^	143.08 ± 3.96a	142.41 ± 5.59a
AP (mg kg^−1^)	Bulk soil	17.40 ± 1.66d	49.13 ± 1.37c	57.77 ± 1.20b^*^	68.40 ± 1.44a^*^
	Rhizosphere	19.27 ± 1.50d	54.90 ± 0.78b^*^	50.37 ± 0.71c	58.33 ± 1.23a
AK (mg kg^−1^)	Bulk soil	146.47 ± 1.05b	143.73 ± 2.96b	168.65 ± 1.69a	163.00 ± 5.96a
	Rhizosphere	218.40 ± 46.05ab	191.44 ± 1.55b^*^	246.39 ± 8.77a^*^	251.03 ± 7.14a^*^
Soybean yield (kg ha^−1^)		2287.60 ± 119.86b	2856.67 ± 160.32a	2881.33 ± 173.27a	3025.33 ± 131.37a

Soil properties were calculated for each replicate of fertilizer treatment and bulk soil/rhizosphere samples (*n* = 3). Data are the means ± standard deviation. CK: non-inoculated control in soil; PK, superphosphorus and potassium chloride; PK + N, PK chemical fertilizers plus urea; PK + R, PK chemical fertilizers plus *Bradyrhizobium japonicum* 5821. Different letters in each column of same fertilization treatment indicate significant difference among treatments in different stage (*p* < 0.05). The asterisks indicate the significance of the difference between the indexes of bulk and rhizosphere soil under the same treatment during the same growth stage (*p* < 0.05).

### 3.2. Variations in abundance and richness of bacteria

**Figure 1:**
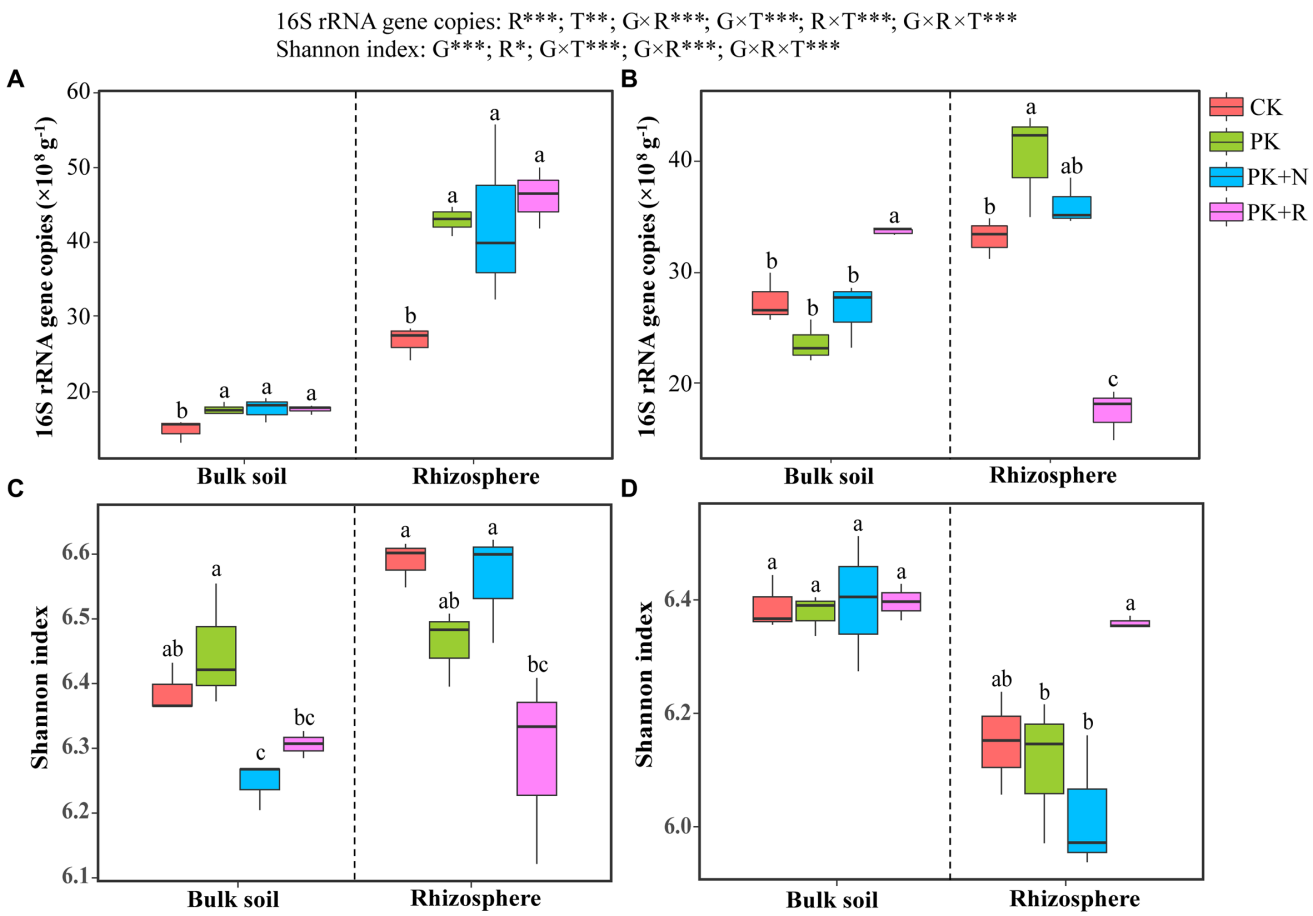
Bacterial abundance **(A,B)** and richness **(C,D)** in soybean field trials at the flowering–podding stage **(A,C)** and maturity stage **(B,D)**. CK: non-inoculated control in soil; PK, superphosphorus and potassium chloride; PK + N, PK chemical fertilizers plus urea; PK + R, PK chemical fertilizers plus *Bradyrhizobium japonicum* 5821. Different letters above bars indicate significant differences (one-way ANOVA, *p* < 0.05, Duncan’s multiple-range test) among different treatments at each growth stage. The overall effects of growth stage (G), rhizosphere effect (R), and treatment (T) on bacterial abundance and Shannon index were evaluated by three-way ANOVA, with the results shown at the top of the figure. *0.01 < *p* ≤ 0.05, **p ≤ 0.01, ***p ≤ 0.001.

Results of the three-way ANOVA confirmed that the rhizosphere effect (*p* < 0.001) and treatment (*p* < 0.01) had remarkable influence on 16S rRNA gene abundance, whereas growth stage had no remarkable influence (Figure 1). The interactions among growth stage, rhizosphere effect, and treatment were noteworthy (*p* < 0.001). The abundances of soil bacteria were 14.91× 10^8^ to 46.07 × 10^8^ copies g^−1^, which in CK were obviously less than other treatments at the flowering–podding stage (Figure 1A), whereas those in PK + R were apparently greater than other treatments in bulk soil and obviously less than other treatments in rhizosphere soil at the maturity stage (Figure 1B).

A complete of 7,132,055 high-quality reads had been obtained from 48 soil samples. The coverage values ranged from 96.05% to 96.52%. Three-way ANOVA confirmed that the growth stage (*p* < 0.001) and rhizosphere effect (*p* < 0.05) had obvious significance on the Shannon index, whereas the treatment had no remarkable influence. The interactions among growth stage, rhizosphere effect, and treatment were noteworthy (*p* < 0.05; Figure 1). At the flowering–podding stage, the Shannon index in the PK treatment was significantly higher than that in the PK + N treatment in the bulk soil, and that in CK and PK + N were greater than other treatments in the rhizosphere soil (Figure 1C). At maturity, there was no remarkable difference in Shannon index among any treatments in the bulk soil. In the rhizosphere soil, Shannon index in PK + R was 2.91%, 5.05%, and 5.05% higher than those in CK, PK, and PK + N, respectively (Figure 1D).

**Figure 2:**
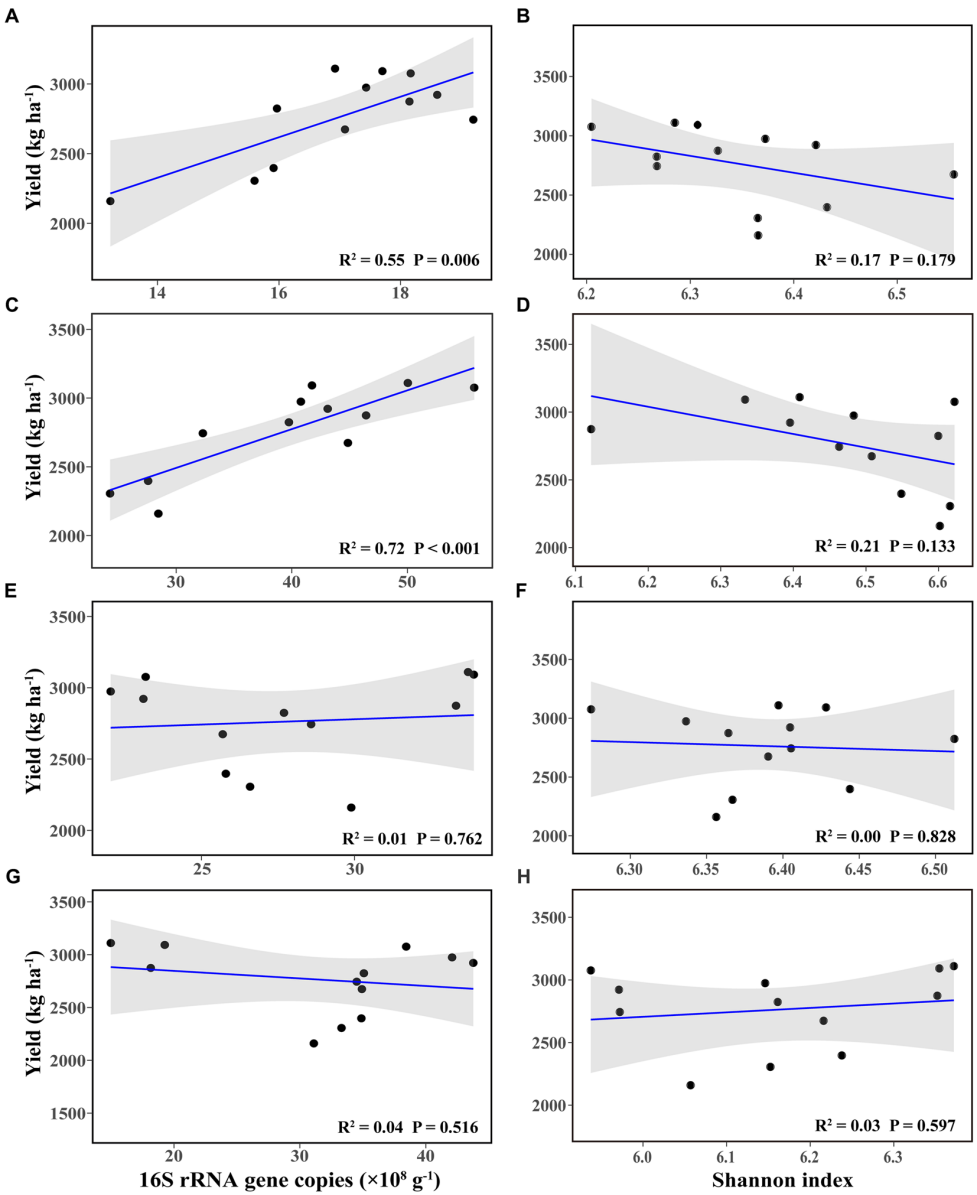
Linear regression relationships between bacterial abundance **(A,C,E,G)** and richness **(B,D,F,H)**, and soybean yield, in bulk **(A,B,E,F)** and rhizosphere soil **(C,D,G,H)**, at the flowering–podding stage **(A–D)** and at the maturity stage **(E–H)**.

Linear regression analysis showed that bacterial abundance in bulk and rhizosphere soil at the flowering–podding stage was positively correlated with soybean yield (Figures 2A,C), whereas they had no significant correlation with soybean yield at the maturity stage (Figures 2E,G). The bacterial richness in bulk and rhizosphere soil at both stages was not correlated with soybean yield (Figures 2B,D,F,H). There was no significant correlation between soybean yield and root nodule dry weight (Supplementary Figure S1).

### 3.3. Variations in community composition of bacteria

**Figure 3:**
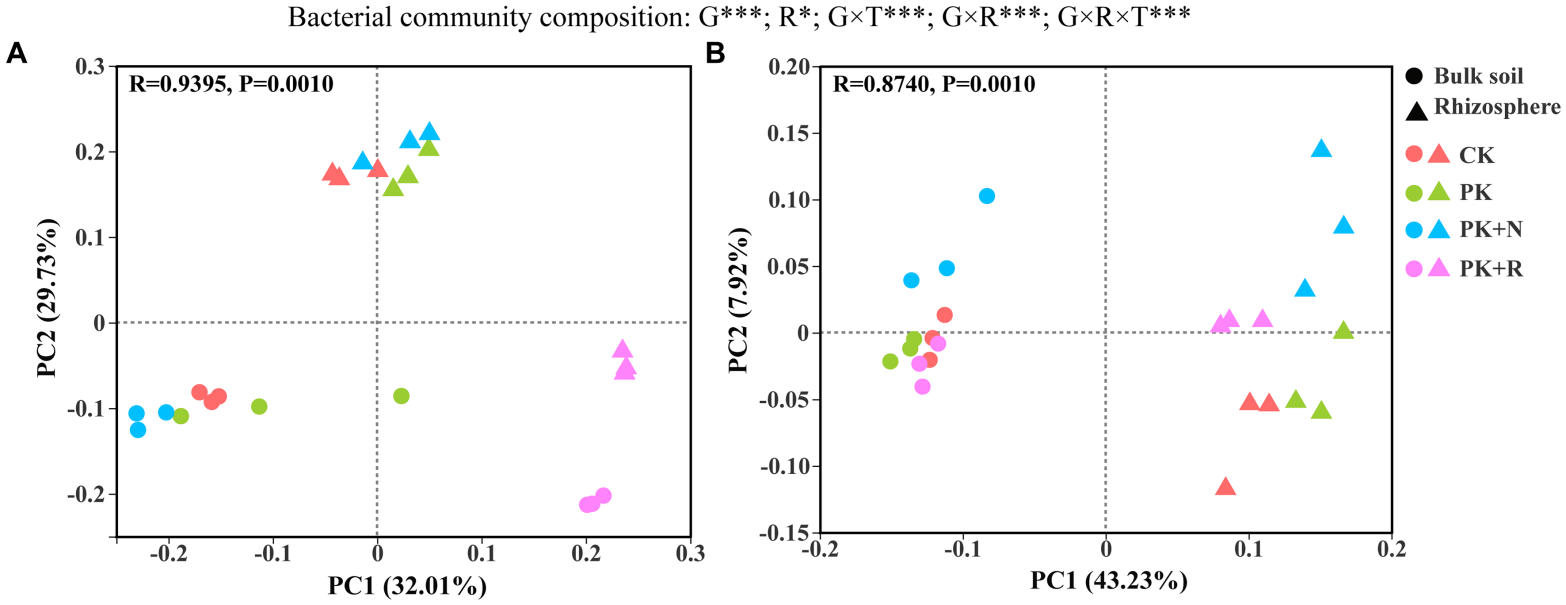
Principal coordinate analysis (PCoA) ordinations of bacterial community composition from the bulk soil and the rhizosphere of soybean under different fertilization levels at the flowering–podding stage **(A)** and the maturity stage **(B)**. Differences in bacterial beta diversity among different fertilization treatments were determined through PERMANOVA based on the Bray–Curtis distance matrix. CK: non-inoculated control in soil; PK, superphosphorus and potassium chloride; PK + N, PK chemical fertilizers plus urea; PK + R, PK chemical fertilizers plus *Bradyrhizobium japonicum* 5821. The effects of growth stage (G), rhizosphere effect (R), and treatment (T) on bacterial community composition were evaluated by three-way ANOVA, with the results shown at the top of the figure. *0.01 < *p* ≤ 0.05, **p ≤ 0.01, ***p ≤ 0.001.

PCoA analysis of the bacterial community structure showed that there was a significant separation among the clusters at both growth stages (*p* = 0.001; Figure 3). At the flowering–podding stage, 32.01% could be explained by PC1 and 29.73% by PC2 (Figure 3A). Meanwhile, at the maturity stage, 43.23% could be explained by PC1 and 7.92% by PC2 (Figure 3B). The different treatments presented remarkable separation at the flowering–podding and maturity stages. Rhizosphere effects also led to prominent separation of the bacterial community structure (Figures 3A,B). Three-way ANOVA showed that the growth stage (*p* < 0.001) and rhizosphere effect (*p* < 0.05) had significant influence on the bacterial community composition, whereas the treatment had no remarkable influence. The effects of different treatments on bacterial beta diversity were significantly different at the same stage and space scale (*p* = 0.001, Supplementary Table S1). Specifically, at the flowering-podding stage, the *R*^2^ value was 0.7963 in bulk soil and 0.9506 in rhizosphere soil. At the maturity stage, the *R*^2^ value was 0.7037 in bulk soil and 0.7562 in rhizosphere soil.

**Figure 4:**
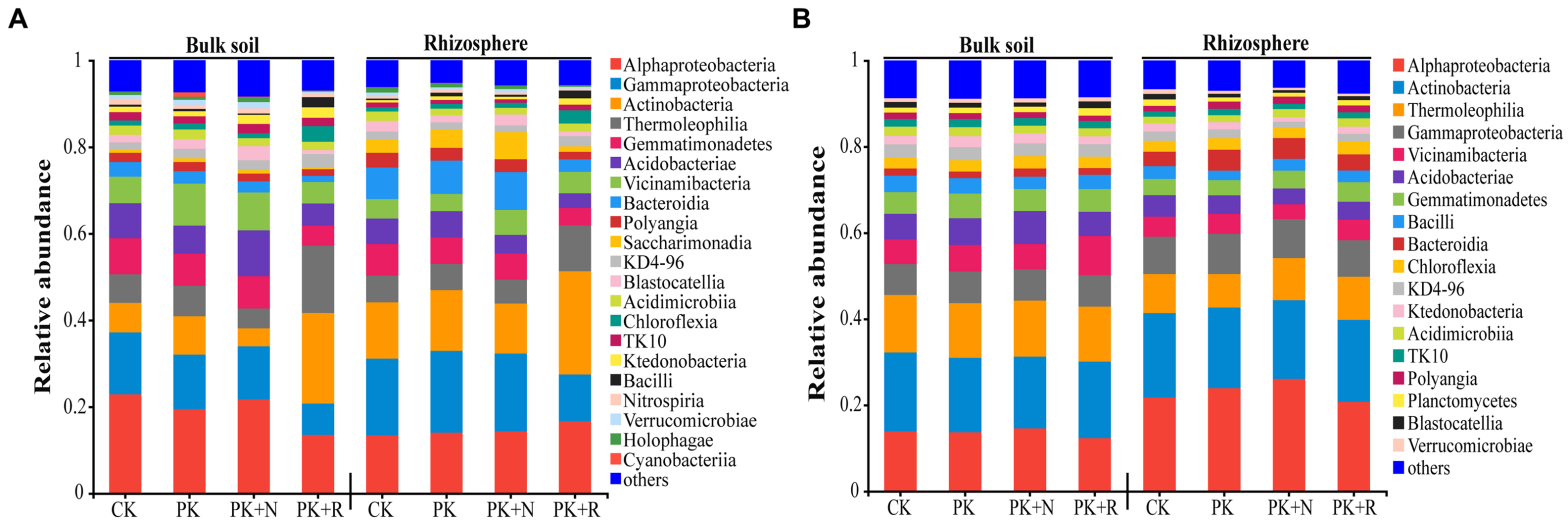
Relative abundance of dominant bacteria at the class level (relative abundance >1%) **(A,B)** for each treatment at the flowering–podding stage **(A)** and at the maturity stage **(B)**. CK: non-inoculated control in soil; PK, superphosphorus and potassium chloride; PK + N, PK chemical fertilizers plus urea; PK + R, PK chemical fertilizers plus *Bradyrhizobium japonicum* 5821.

The predominant bacterial classes in all samples were Alphaproteobacteria, Actinobacteria, Thermoleophilia, Gammaproteobacteria, Acidobacteria, Vicinamibacteria, and Gemmatimonadetes, accounting for 65.44–74.24% of the total sequences at the flowering–podding stage (Figure 4A) and 69.11–74.42% at the maturity stage (Figure 4B). Subsequently, we analyzed the changes in dominant bacteria communities at class level caused by fertilization and rhizobium (Supplementary Table S2). Notably, the application of the same fertilizer or rhizobium in bulk or rhizosphere soil during different growth periods resulted in different dominant bacteria in the community. For instance, when applicated with PK + R, in bulk soil at the flowering–podding stage, the relative abundances of the classes Thermoleophilia, Chloroflexia, and Bacilli were significantly (*p* < 0.05) increased, while the relative abundance of the classes Alphaproteobacteria, Gammaproteobacteria, Gemmatimonadetes, Bacteroidia, Saccharimonadia, and Holophagae were significantly decreased. In rhizosphere soil at the flowering–podding stage, the relative abundances of the classes Actinobacteria, Thermoleophilia, Chloroflexia, Ktedonobacteria, and Bacilli were significantly (*p* < 0.05) increased, while the relative abundance of the classes Gammaproteobacteria, Gemmatimonadetes, Bacteroidia, Polyangia, Saccharimonadia, and Holophagae were significantly decreased. In bulk soil at the maturity stage, the relative abundance of the class Bacteroidia and Gemmatimonadetes was significantly decreased. In rhizosphere soil at the maturity stage, the relative abundances of the classes Alphaproteobacteria, Gammaproteobacteria, and Bacteroidia were significantly (*p* < 0.05) increased, while the relative abundance of the classes Thermoleophilia, and Gemmatimonadetes were significantly decreased. The relative abundance of the class Gemmatimonadetes was significantly decreased in all treatments during the both growth stages.

**Figure 5:**
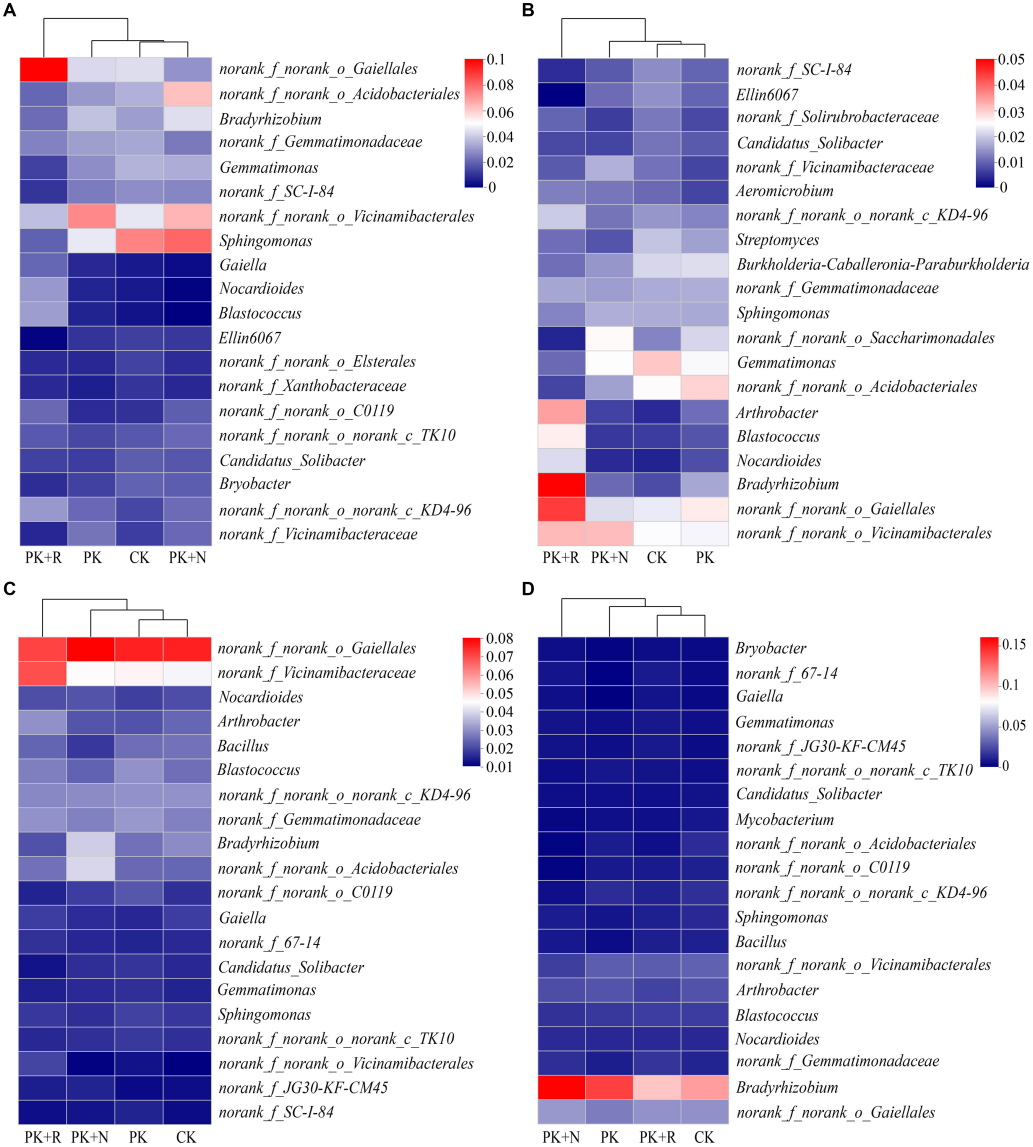
Relative abundances of top 20 dominant genera in different treatments at flowering–podding **(A,B)** and maturity **(C,D)** stages in bulk **(A,C)** and rhizosphere **(B,D)** soil. CK: non-inoculated control in soil; PK, superphosphorus and potassium chloride; PK + N, PK chemical fertilizers plus urea; PK + R, PK chemical fertilizers plus *Bradyrhizobium japonicum* 5821.

The dominant genus was *norank_f_norank_o_Gaiellales* (2.51–7.71%) in bulk and rhizosphere soil at both stages, and their relative abundances varied significantly across different treatments (Figure 5). When applicated with PK + R, in bulk soil at the flowering–podding stage, the relative abundances of the genera *norank_f_norank_o_Gaiellales*, *Gaiella*, *Nocardioides*, and *Blastococcus* were significantly (*p* < 0.05) increased, while the relative abundances of the genera *Gemmatimonas*, *norank_f_SC-I-84*, *Sphingomonas*, and *Ellin6067* were significantly decreased. In rhizosphere soil at the flowering–podding stage, the relative abundances of the genera *Blastococcus*, *Nocardioides*, and *norank_f_norank_o_Gaiellales* were significantly (*p* < 0.05) increased, while the relative abundance of the genera *norank_f_SC-I-84*, *Ellin6067*, *norank_f_norank_o_Saccharimonadales*, and *Gemmatimonas* were significantly decreased. In bulk soil at the maturity stage, the relative abundances of the genera *norank_f_norank_o_Vicinamibacterales*, *Arthrobacter*, and *norank_f_Vicinamibacteraceae* were significantly (*p* < 0.05) increased. Overall, co-application of fertilization and rhizobium significantly increased the relative abundance of the genera *norank_f_norank_o_Gaiellales*, *Nocardioides*, and *Blastococcus*, and decreased the relative abundance of *Gemmatimonas*, *norank_f_SC-I-84*, and *Ellin6067* in bulk and rhizosphere soil at the flowering–podding stage. In addition, fertilization resulted in a significant reduction of *norank_f_norank_o_Elsterales* and *norank_f_Xanthobacteraceae* in bulk soil at flowering–podding stage. Application of nitrogen fertilizer resulted in significant decrease in *Bacillus* in bulk soil, and *Mycobacterium* and *norank_f_norank_o_C0119* in rhizosphere soil at maturity stage (Figure 5; Supplementary Table S3). The relative abundances of *Bradyrhizobium* in bulk and rhizosphere soil at the flowering–podding and maturity stages are shown in Supplementary Figure S2. Specifically, the relative abundance of *Bradyrhizobium* was lower in PK + R compared to PK + N in bulk soil at both the flowering-podding and maturity stages. However, in rhizosphere soil at the flowering-podding stage, the relative abundance of *Bradyrhizobium* was higher in PK + R compared to PK + N, while the opposite trend was observed at the maturity stage.

**Figure 6:**
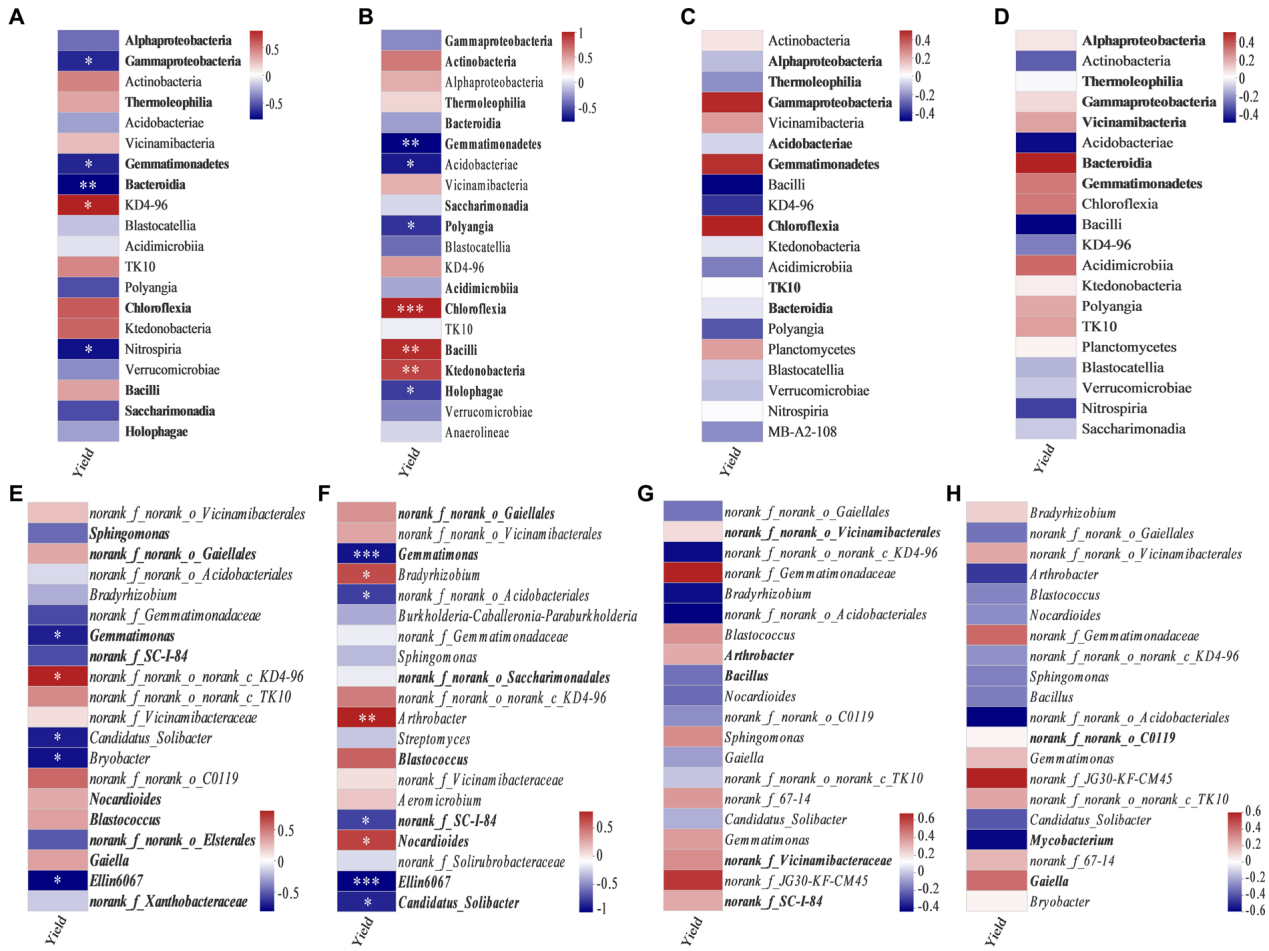
Spearman correlation coefficients between soybean yield and dominant bacteria at class (relative abundance >1%) **(A–D)** and genus level (20 most abundant) **(E–H)** in bulk soil **(A,C,E,G)** and rhizosphere soil **(B,D,F,H)** at the flowering–podding stage **(A,B,E,F)** and maturity stage **(C,D,G,H)**. Bold font indicates classes and genera with significant differences among different treatments. *0.01 < *p* ≤ 0.05, **p ≤ 0.01, ***p ≤ 0.001.

Spearman correlation analysis revealed the correlation between soybean yield and major bacterial communities at class and genus levels (Figure 6). In bulk soil, at the flowering–podding stage, the class KD4–96 (Figure 6A) and the genus *norank_f_norank_o_norank_c_KD4-96* (Figure 6E) were positively correlated with soybean yield. Conversely, the classes Gammaproteobacteria, Gemmatimonadetes, Bacteroidia, and Nitrospiria (Figure 6A), and the genera *Gemmatimonas*, *Candidatus*_*Solibacter*, *Bryobacter*, and *Ellin6067* were negatively correlated with soybean yield (Figure 6E). The bacterial communities in rhizosphere soil at the flowering–podding stage showed that the relative abundances of the classes Chloroflexia, Bacilli, and Ktedonobacteria (Figure 6B), and the genera *Bradyrhizobium*, *Arthrobacter*, and *Nocardioides* (Figure 6F) were positively correlated with soybean yield. The classes Gemmatimonadetes, Acidobacteria, Polyangia, and Holophagae (Figure 6B), and the genera *Gemmatimonas*, *norank_f_norank_o_Acidobacteriales*, *norank_f_SC-I-84*, *Ellin6067*, and *Candidatus*_*Solibacter* (Figure 6F) were negatively correlated with soybean yield. In summary, the significant different class Gemmatimonadetes, and the genera *Gemmatimonas* and *Ellin6067* in soil at the flowering–podding stage were negatively correlated with soybean yield. However, the soil bacterial community at both class and genus levels at maturity was not correlated with soybean yield (Figures 6C,D,G,H).

### 3.4. Factors driving bacteria variation in black soil

**Figure 7:**
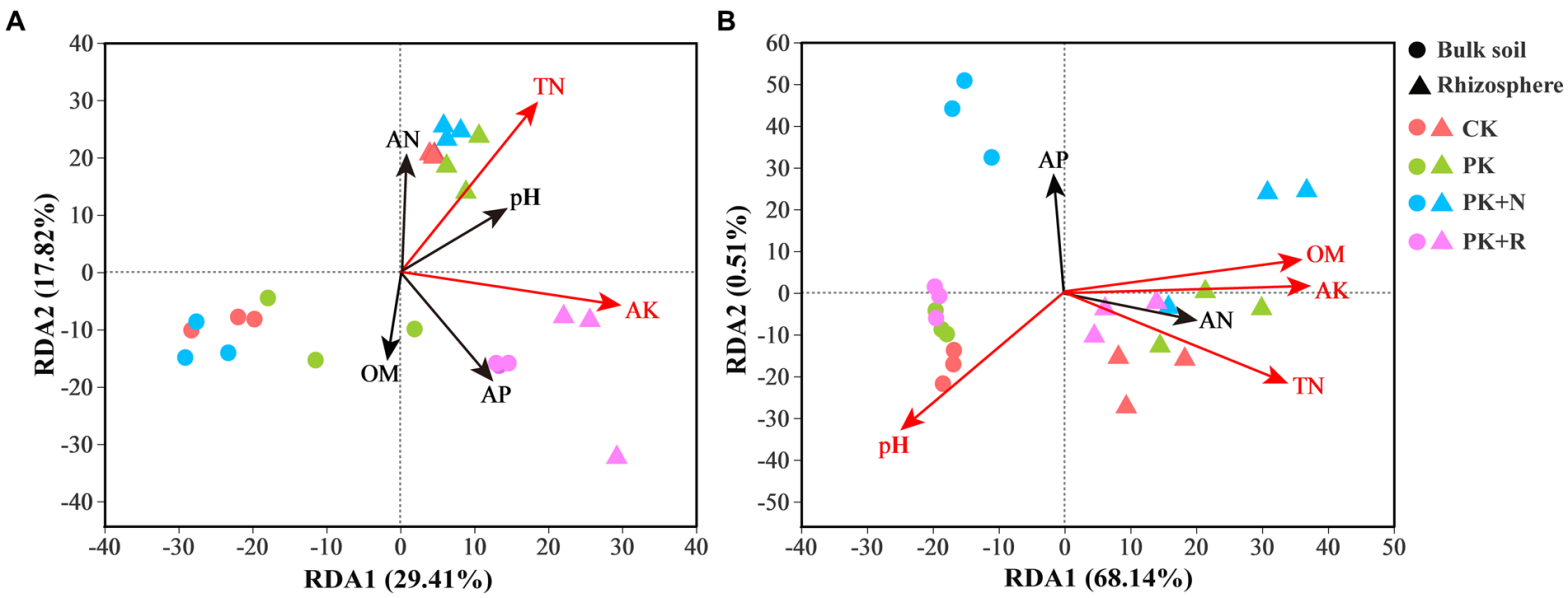
Redundancy analysis (RDA) profile constructed from the OTU composition of bacteria and soil properties in the bulk and rhizosphere soil samples under different fertilization levels at the flowering–podding stage **(A)** and the maturity stage **(B)**. The position and length of the arrows indicate the direction and strength of the influence of soil variables on bacterial communities, respectively. The significant variables are shown by red arrows (*p* < 0.01). CK, non-inoculated control in soil; PK, superphosphorus and potassium chloride; PK + N, PK chemical fertilizers plus urea; PK + R, PK chemical fertilizers plus *Bradyrhizobium japonicum* 5821.

The RDA analysis showed the effects of soil properties on bacterial community composition at different stages (Figure 7). Overall, 29.41% of variation in the bacterial composition in these treatments at the flowering–podding stage was explained by RDA1 and 17.82% by RDA2 (Figure 7A). At the maturity stage, 68.14% of variation was explained by RDA1 and 0.51% by RDA2 (Figure 7B). Treatments at both stages in the bulk soil were separated along RDA2, whereas the treatments in rhizosphere soil were isolated from the bulk soil along RDA1. AK and TN were significantly related to bacterial communities at flowering-podding stage, and AK, OM, TN, and pH at maturity stage. This indicates the presence of a specific bacterial community composition between the bulk and rhizosphere soil treatments. Mantel test was proceeded to further investigate the influence of environmental factors on bacterial composition. There was a significant positive correlation between pH and bacterial community in the rhizosphere soil at the maturity stage (*p* < 0.05, Supplementary Table S4).

### 3.5. Integrated responses of soil properties and soybean yield on bacterial structure

Spearman’s correlation analyses between soil properties, nodule dry weight, soybean yield, and bacterial structure are shown in Supplementary Table S5. Nodule dry weight was negatively related to pH and positively related to AP and AK contents. Soybean yield was positively related to AP. Bacterial abundance was positively related to AN and AK, whereas bacterial diversity was negatively related to OM, AN, and AK. Bacterial composition was positively related to OM, AN, and AK, and negatively related to TN and bacterial abundance.

**Figure 8:**
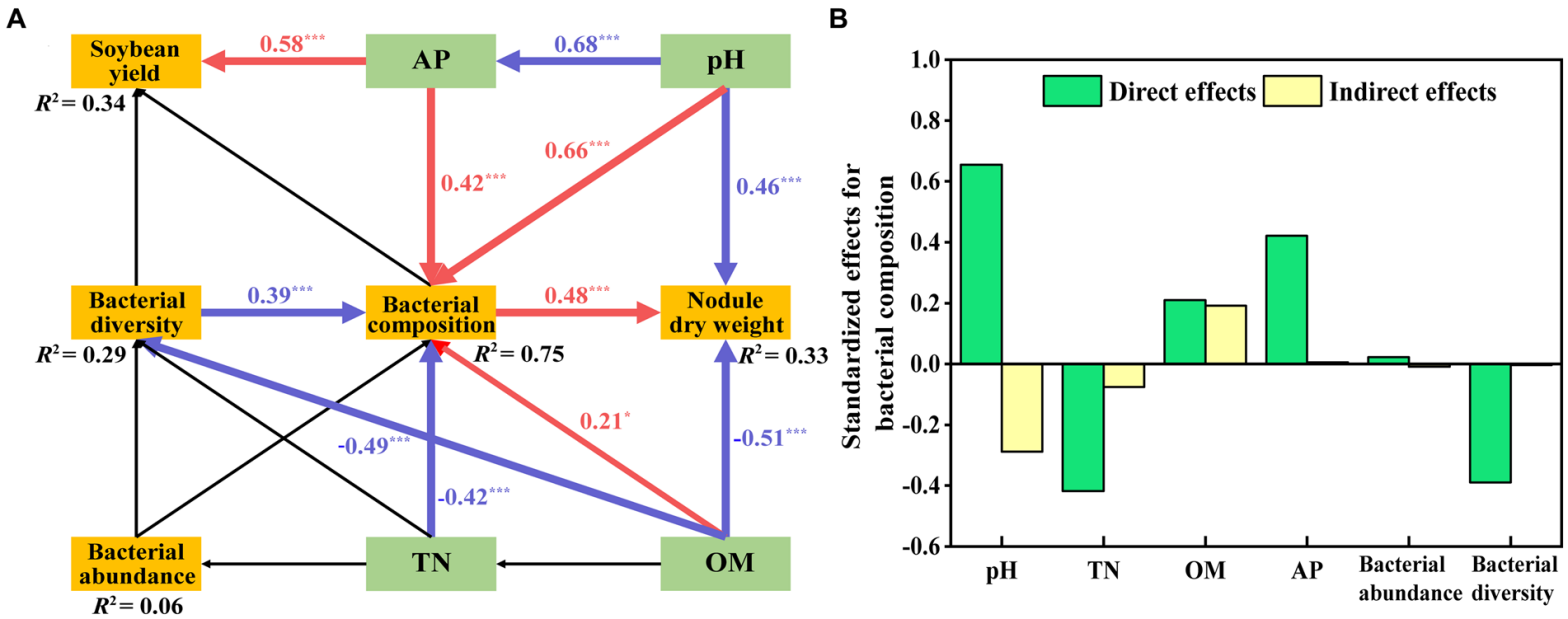
Structural equation model (SEM) showing the hypothesized causal relationships among soil properties (OM, TN, pH, AP), nodule dry weight, soybean yield and bacterial abundance, diversity, and composition **(A)**. This model resulted in a good fit to the data, with a model *χ*^2^ = 24, df = 18, *p* = 0.16, GFI = 0.92, RMSEA = 0.08. Red arrows indicate significant positive correlations, while blue indicates significant negative relationships (*p* < 0.05). *R*^2^ values represent the proportion of the variance explained for each endogenous variable. The direct and indirect effects of factors on bacterial composition were determined using SEM **(B)**.

We assessed the effects of bacterial structure on main physicochemical factors and the soybean yield by using an SEM model based on Supplementary Table S5 (Figure 8A). This model fits our causal hypothesis (*χ*^2^ = 24, df = 18, *p* = 0.16, GFI = 0.92, RMSEA = 0.08). The effect of AP (path coefficient = 0.58) on soybean yield was significantly positive. The path coefficient of pH on bacterial composition was 0.66, which was higher than that of AP (0.42) and OM (0.21). The negative path coefficient of TN on bacterial composition was −0.42, which was higher than the path coefficient on bacterial diversity (−0.39). Bacterial composition positively affected nodule dry weight, and the path coefficient was 0.48, whereas OM and pH negatively affected nodule dry weight, the path coefficient was −0.51 and − 0.46. The final model explained the weightiness of different components, with soybean yield accounting for 34%, nodular dry weight for 33%, bacterial abundance for 6%, bacterial composition for 75%, and bacterial diversity for 29%. Details of the standardized direct and indirect effects for the SEM models are shown in Figure 8B.

## 4. Discussion

### 4.1. Long-term co-application of *Bradyrhizobium japonicum* 5821 and fertilizer promotes soybean yield and alters soil properties

Rhizobium inoculation has been suggested to promote soybean nodules and yields (Albareda et al., 2009; Hungria et al., 2013; Zhong et al., 2019; Ulzen et al., 2020). In our study, long-term inoculation with *B. japonicum* 5821 and application of PK fertilizer promoted soybean nodule dry weight by 33.94% when compared with PK + N, and increased soybean yield when compared with CK, PK, and PK + N (Table 1). This was similar to the study, soybean yield increased by 10.1% (180 kg hm^−1^) after 8 years of prolonged field inoculation with *Bradyrhizobium* and NPK fertilizer application in central India (Rawat et al., 2013). However, most previous studies have been carried out on other crops based on short-term field trials, not at long-term scale. For example, 2 years of field experiments showed that inoculating rhizobia alone was not enough to promote cowpea yield; only rhizobia inoculation combined with phosphate and potassium fertilizer could promote the yield (Chiamaka, 2015). It can be seen that long-term inoculation could increase soybean yield, but short-term inoculation did not necessarily increase yield, which was related to the cumulative effects of continuous inoculation, suggesting that continuous inoculation was necessary for production. In addition, *Bradyrhizobium* spp. inoculation and N fertilizer application for two consecutive years increased soybean yield by 130 kg ha^−1^, and promoted soybean dry weight and nitrogen content, but N fertilizer application without rhizobia inoculation only increased plant dry weight, the effect on soybean yield and nitrogen content was insignificant (Ruiz Diaz et al., 2009). Consequently, rhizobium combined with appropriate fertilizer can improve the yield of leguminous crops by improving soil fertility and crop root environment, and providing sufficient nitrogen for crop growth (Quandt and Hynes, 1993; Nett et al., 2022). And *B. japonicum* 5821 inoculation instead of nitrogen fertilizer increased soybean yield.

Long-term nitrogen fertilization (PK + N) reduced root nodule dry weight by 69% when compared with PK, and by 33.94% when compared with PK + R, because soil biological nitrogen fixation can be inhibited by excessive nitrogen fertilizer (Liu et al., 2019; Smercina et al., 2019; Zheng et al., 2019). Excessive nitrogen application can also negatively affect soybean growth (Zhou et al., 2006). Nitrogen application weakened the symbiotic nitrogen fixation ability of soybean with different genotypes (Reinprecht et al., 2020). Nitrogen fertilizer significantly reduced rhizobium abundance treated by ambient CO_2_ (Liu et al., 2021). In addition, PK + R had higher soybean yield and nodulation compared with PK + N. The synergistic effects of nodulation and nitrogen fixation on yield indicated that rhizobium inoculation reduced nitrogen availability, this is in line with preceding research (Turan et al., 2019; Sanyal et al., 2020). Therefore, rhizobia inoculation is a greener and more effective agricultural management measure than nitrogen fertilizer application.

The difference of pH between bulk and rhizosphere soil is insignificant in each treatment at the flowering–podding stage, which confirmed the cumulative effect and precise effect of long-term targeted inoculation of rhizobia. Long-term fertilization resulted in soil acidification (Barak et al., 1997; Schroder et al., 2011; Yang et al., 2018). Our study also showed that long-term fertilization resulted in a significant decrease in soil pH (Table 1). The pH of PK + R was obviously greater than that of PK and PK + N at the flowering–podding stage, suggesting that inoculation with *B. japonicum* 5821 can prevent soil acidification. This observation is consistent with preceding research (Watkin et al., 2000; Makoi et al., 2013; Alemayehu and Dechassa, 2022). Because rhizobia was more suitable for survival in near neutral pH environment than low pH environment. Inoculating rhizobium significantly increased soil pH, further increased the availability of Ca, Na, Fe, Cu, Zn, and Mn nutrients in rhizosphere soil (Bambara and Ndakidemi, 2010), also considerably extended the absorption of nutrient elements such as P, K, Ca, and Mg in plants (Makoi et al., 2013). It is suggested that inoculation with rhizobium inhibits soil acidification by increasing nutrient availability in soil and extending absorption of nutrient in plants. The total and available nutrients of rhizosphere soil were higher than that of bulk soil at the maturity stage, which is supported by recent studies (Chen et al., 2018, 2019; Li et al., 2019). This may be related to the carbohydrate and amino acid substances in the rhizosphere secretions promoted the contents of various nutrients in the rhizosphere soil, resulted in a significantly different rhizosphere microenvironment from that in bulk soil (Ai et al., 2013). Furthermore, the pH of PK + R was observably less than that of CK at the flowering–podding stage, indicating certain limitations on the effect of rhizobia inoculation on soil pH. Thus, inoculation with rhizobia can prevent soil acidification to some extent.

### 4.2. Effects of long-term co-application of *Bradyrhizobium japonicum* 5821 and fertilizer on bacterial community composition

Rhizobia inoculation not only affected the growth of aboveground crops, but also soil microbiota. In the present study, a combination of amplicon sequencing and qPCR analyses revealed that fertilization and inoculation with rhizobia had far-reaching influence on bacterial abundance and richness (Figure 1). We observed that at the maturity stage, bacterial abundance (in bulk soil) and richness (in rhizosphere soil) of PK + R were significantly higher than those of other treatments, indicating that co-application of *B. japonicum* 5821 and PK chemical fertilizers increased bacterial abundance and richness. This is consistent with previous reports on single inoculation of rhizobia or single fertilization. Fall et al. (2016) observed that rhizobium inoculated with native trees of *Senegalia senegal* (L.) Britton in a gum Arabic production area improved soil microbial biomass and functional diversity. Phaseolus beans inoculated with two native rhizobium significantly increased bacterial richness (Trabelsi et al., 2011). Co-inoculation of alfalfa with rhizobia slightly promoted microbial diversity in rhizosphere soil (Ju et al., 2019). Wang et al. (2018) characterized rhizosphere and bulk soil bacterial communities in a 36-year fertilizer experiment and found that application of N fertilizer decreased bacterial abundance, whereas MNPK (horse plus NPK) fertilizer enhanced bacterial abundance in the maize soil. Long-term application of high phosphorous fertilizer reduced bacterial diversity in wheat rhizosphere soil (Liu et al., 2020). Liu et al. (2021) reported that inoculation with *Bradyrhizobium diazoefficiens* USDA 110 reduced the diversity of soybean rhizosphere microbes. These inconsistent results may be on account of the interaction between soil and plants, which leads to complex soil environments and diverse microorganisms (Wu et al., 2011; Francioli et al., 2016; Hu et al., 2018; Semenov et al., 2020). Furthermore, the bacterial abundance in PK + R was observably lower than in other treatments in rhizosphere soil at maturity (Figure 1B). First, this may be because the inoculation of rhizobia inhibits the growth of other bacteria, leading to the enrichment of rhizobia in the soybean rhizosphere soil. Secondly, rhizobium can symbiosis nitrogen fixation with soybean, and soybean growth slows down after entering the maturity stage compared with the earlier stages, resulting in a decrease in bacterial abundance (Sohn et al., 2021). Third, crop roots mainly stimulate specific rhizosphere groups, resulting in communities that become increasingly different from the bulk soil, often with lower diversity (Shi et al., 2015; Fan et al., 2017; Nuccio et al., 2020).

Co-application of *B. japonicum* 5821 and PK fertilizer led to significant diversification of soil bacterial community composition (Supplementary Tables S2, S3). The co-application decreased the relative abundance of the class Gemmatimonadetes in all treatments during both growth stages (Figure 4); and significantly increased the relative abundance of the genera *norank_f_norank_o_Gaiellales*, *Nocardioides*, and *Blastococcus*, and decreased the relative abundance of *Gemmatimonas*, *norank_f_SC-I-84*, and *Ellin6067* in bulk and rhizosphere soil at the flowering–podding stage (Figure 5). Such variation in bacterial composition caused by long-term inoculation was similar to those in the short-term scale. Sun et al. (2009) carried out 1-year field inoculation experiments, found that inoculation with *Sinorhizobium meliloti* CCBAU01199 increased Alphaproteobacteria and Betaproteobacteria relative abundance, decreased Gammaproteobacteria, Deltaproteobacteria, Firmicutes, and Actinobacteria abundance in alfalfa rhizosphere soil. During in situ restoration of a vanadium titanomagnetite tailings dam using *Pongamia pinnata* for 2 years, the abundance of groups under the phylum Proteobacteria increased in rhizosphere flora, and OTUs associated with rhizobia were preferably enriched (Yu et al., 2019). Furthermore, fertilization has been shown to alter soil microbial community composition (Geisseler and Scow, 2014; Ma et al., 2018; Zhou et al., 2019; Zhu et al., 2021). Nitrogen directly affect the bacterial community composition and soil factors, NPK directly affect the fungi community composition (Cassman et al., 2016). We can imply that long-term co-application of *B. japonicum* 5821 and PK fertilizer induced varied changes in the bacterial community structures (Zhu et al., 2018; Chee-Sanford et al., 2019; Kalam et al., 2022).

From our results, soil bacterial abundance at the flowering–podding stage were positively correlated with soybean yield, but not at the maturity stage (Figure 2). This is related to the different ecological functions of bacterial community in plant development stage. Root exudates are the communication link between plants and soil bacterial communities. Plants at different development stages release root exudates to change the assembly of plant microbiome (Ajilogba et al., 2022). And soil microbial activity at the flowering–podding stage of soybean was more vigorous than that at the maturity stage (Xu et al., 2009; Sohn et al., 2021). Additionally, Spearman correlation results revealed that at the flowering–podding stage, the class KD4–96 and the genus *norank_f_norank_o_norank_c_KD4-96* in bulk soil, the classes Chloroflexia, Bacilli, and Ktedonobacteria, and the genera *Bradyrhizobium*, *Arthrobacter*, and *Nocardioides* in rhizosphere soil, were positively correlated to soybean yield. The significant different class Gemmatimonadetes, the genera *Gemmatimonas* and *Ellin6067* in bulk and rhizosphere soil were negatively correlated with soybean yield (Figures 6A,B,E,F). The results showed that these bacteria had significant effect on soybean yield. This was similar to the report by Niraula et al. (2022) who observed that the rhizosphere soil microorganisms during soybean R1 (beginning of flowering)—R2 (blooming) stages, the class Anaerolineae, family Micromonosporaceae, and genera *Plantomyces*, *Nitrospira*, and *Rhizobium* have important effects on soybean yield. But the soil bacterial community at both class and genus levels at maturity was not significantly correlated with soybean yield (Figures 6C,D,G,H), this further indicated that the key bacterial communities determine soybean yield were concentrated in the early stages of soybean growth.

Mantel test and Spearman correlation results showed that soil OM, TN, pH, and AP were the dominant variables affecting bacterial community composition (Supplementary Tables S4, S5). The SEM showed significant effects of the four variables and bacterial diversity on bacterial composition. Nodule dry weight was negatively affected by OM and pH, and soybean yield was positively affected by AP (Figure 8). Preceding reports agree with our results (Yan et al., 2014, 2019; Liu et al., 2021).

## 5. Conclusion

In the present study, we evaluated the effects of four treatments (CK, PK, PK + N, and PK + R) on the bacterial composition of soybean grown in the black soil of Northeast China at the flowering–podding and maturity stages. Long-term inoculation with *B. japonicum* 5821 and application of PK fertilizer increased soybean nodule dry weight and soybean yield and altered soil properties. Co-application of *B. japonicum* 5821 and PK increased bacterial abundance in soybean bulk soil, and reduced bacterial abundance in rhizosphere soil at the maturity stage. The classes Alphaproteobacteria, Actinobacteria, Thermoleophilia, Gammaproteobacteria, Acidobacteria, Vicinamibacteria, and Gemmatimonadetes were the dominant bacteria across all the soil samples. Co-inoculation with *B. japonicum* 5821 and PK fertilizer strongly altered the bacterial community composition. The key bacterial communities that determine soybean yield were concentrated in the flowering–podding stage, not at maturity stage. Soil OM, TN, pH, and AP were the dominant variables affecting bacterial composition. The results demonstrate that long-term inoculation of rhizobia has the potential to promote soybean productivity and nitrogen fixation ability, and to improve soil fertility.

## Data availability statement

The datasets presented in this study can be found in online repositories. The names of the repository/repositories and accession number(s) can be found at: NCBI—PRJNA859097.

## Author contributions

WW, MM, XJ, and JL designed the study. FM provided resources. WW, DG, LL, YZ, FC, and HC performed the experiments. WW analyzed all the data, prepared the figures and tables, and wrote the first draft of the manuscript. FF, BZ, and JL edited the manuscript and checked the language. All authors contributed to the article and approved the submitted version.

## Funding

This work was supported by the Major Science and Technology Project of Yunnan Province (202202AE090025), the National Key Research and Development Program of China (2021YFD1700200), the Earmarked Fund for Modern Agro-industry Technology Research System (CARS-04), and the National Natural Science Foundation of China (32201320).

## Conflict of interest

The authors declare that the research was conducted in the absence of any commercial or financial relationships that could be construed as a potential conflict of interest.

## Publisher’s note

All claims expressed in this article are solely those of the authors and do not necessarily represent those of their affiliated organizations, or those of the publisher, the editors and the reviewers. Any product that may be evaluated in this article, or claim that may be made by its manufacturer, is not guaranteed or endorsed by the publisher.
