# Supplementary material for: Long-term fertilization coupled with rhizobium inoculation promotes soybean yield and alters soil bacterial community composition
Source: Front Microbiol. 2023 May 18;14:1161983. doi: 10.3389/fmicb.2023.1161983 (PMC10232743; doi:10.3389/fmicb.2023.1161983)
Supplement: Supplementary file 1 [file Data_Sheet_1.docx]

Long-Term Fertilization Coupled with Rhizobium Inoculation Promotes Soybean Yield and Alters Soil Bacterial Community Composition

Wanling Wei^1^, Dawei Guan^1^, Mingchao Ma^1,2^, Xin Jiang^1,2^, Fenliang Fan^1^, Fangang Meng^3^, Li Li^1,2^, Baisuo Zhao^2^, Yubin Zhao^1^, Fengming Cao^1,2^, Huijun Chen^1^, Jun Li^1,2*^

^1^ Institute of Agricultural Resources and Regional Planning, Chinese Academy of Agricultural Sciences, Beijing, China

^2^ Laboratory of Quality & Safety Risk Assessment for Microbial Products (Beijing), Ministry of Agriculture, Beijing, China

^3^ Soybean Research Institute, Jilin Academy of Agricultural Sciences, Jilin, China

* Correspondence: Jun Li
Corresponding Author
lijun01@caas.cn


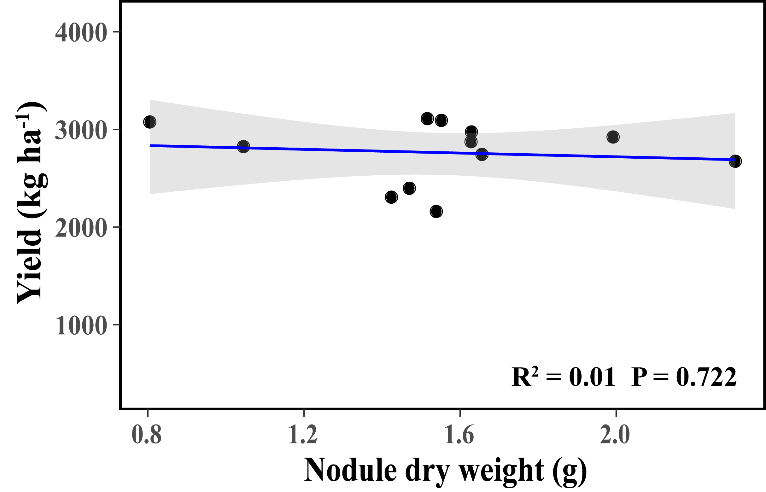


**Figure S1.** Linear regression relationships between nodule dry weight and soybean yield.


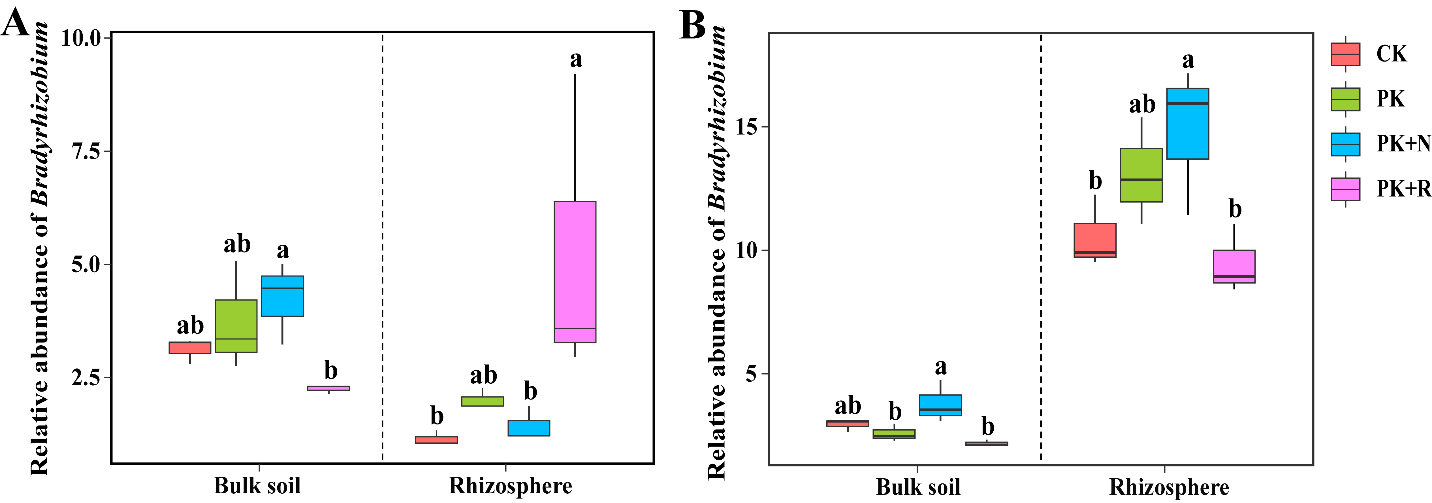


**Figure S2.** The relative abundance of *Bradyrhizobium* in bulk and rhizosphere soil at the flowering–podding (**A**) and maturity (**B**) stages. CK: non-inoculated control in soil; PK, superphosphorus and potassium chloride; PK + N, PK chemical fertilizers plus urea; PK + R, PK chemical fertilizers plus *B. japonicum* 5821. Different letters above bars indicate significant differences among different treatments at each growth stage.
